# Potential Application of *Drosophila melanogaster* as a Model Organism in COVID-19-Related Research

**DOI:** 10.3389/fphar.2020.588561

**Published:** 2020-09-04

**Authors:** Firzan Nainu, Dini Rahmatika, Talha Bin Emran, Harapan Harapan

**Affiliations:** ^1^ Faculty of Pharmacy, Hasanuddin University, Makassar, Indonesia; ^2^ Department of Pharmaceutical Science, Faculty of Mathematics and Natural Sciences, Lambung Mangkurat University, Banjar Baru, Indonesia; ^3^ Department of Pharmacy, BGC Trust University Bangladesh, Chittagong, Bangladesh; ^4^ Medical Research Unit, School of Medicine, Universitas Syiah Kuala, Banda Aceh, Indonesia; ^5^ Tropical Disease Centre, School of Medicine, Universitas Syiah Kuala, Banda Aceh, Indonesia; ^6^ Department of Microbiology, School of Medicine, Universitas Syiah Kuala, Banda Aceh, Indonesia

**Keywords:** SARS-CoV 2, COVID-19, fruit fly, model organism, RNAi screening, *in vivo*, *in vitro*

Since December 2019, the severe acute respiratory syndrome coronavirus 2 (SARS-CoV-2) has been reported to cause a life-threatening malady with multi-spectrum symptoms, called coronavirus disease 2019 (COVID-19). As of August 19, 2020, the disease has spread to more than 225 countries and infected over 20 million people worldwide (Dong et al., 2020), causing upheavals in many aspects of human life (Harapan et al., 2020). At present, much effort has been given to define the clinical characteristics of COVID-19 patients and to discover safe and efficacious vaccines and therapeutics to combat SARS-CoV-2 infection (Dhama et al., 2020). Yet, much more data such as the characteristics of viral genes responsible in the infectivity, factors accountable in the host susceptibility to SARS-CoV-2 infection, viral pathogenesis and the subsequent protective role of host immune responses, and the culmination of host-virus interaction into different types of symptoms in the COVID-19 patients, remains to be fully elucidated. This knowledge is crucial to improve the prevention efforts and treatment strategies needed to manage the spread of COVID-19.

In the presence of ethical constraints to perform explorative research on humans, using proper model organisms to address the above unrequited yet essential aspects of COVID-19 is urgently required. With the aid of suitable animal models, the pharmacodynamics and pharmacokinetic profiles as well as the safety of novel pharmaceuticals to treat COVID-19 can be investigated in a relatively easier, safer, and economical way. Also, identification of the main pathways for COVID-19 pathophysiology that may provide insights for disease prevention and/or treatment can be accomplished in relative terms where possible. Already, several animal models have been used to study SARS-CoV-2 infection. For instance, a study using rhesus macaque, a non-human primate with close phylogenetic proximity to humans, revealed the significance of immunity to protect the host from COVID-19 reinfection (Bao et al., 2020a). While non-human primates represent the strongest ally to solve some COVID-19 enigmatic problems *in vivo*, it is important to note that studies using this model were mostly performed using small numbers of animals in their experiments (e.g., as low as one or two rhesus macaques per group) due to the ethical concerns and animal scarcity. Therefore, the results should be interpreted with caution. To address this issue, several alternative animal models were proposed (Takayama, 2020; Yuan et al., 2020).

Based on its highly characterized immune system, rapid breeding cycle, simplicity, and availability of research tools, mice (*Mus musculus*) have been regarded as one of the most common animal models to demonstrate the pathological properties of viruses and their consequences on the host respiratory system. However, the spike (S) protein of SARS-CoV-2 has been suggested to have an insufficient affinity for the murine ACE2 entry receptor (Wan et al., 2020), which may explain the limitation to obtain a productive infective state in this model. Nevertheless, transgenic mice with endogenous expression of human ACE2 has been developed to address this issue (Bao et al., 2020b). Unfortunately, during the times of COVID-19 pandemic, the low availability of hACE2 transgenic mice might be difficult to compensate for the increasing demand for such a model, thus restraining its extensive use in COVID-19 research (Soldatov et al., 2020).

Since its discovery, some basic information about COVID-19 and its causative agent, SARS-CoV-2, has been rapidly revealed with animal models (Takayama, 2020; Yuan et al., 2020). In this article, we propose using the fruit fly *Drosophila melanogaster* as one of the promising model organisms to unveil specific COVID-19-related questions based on several considerations. First, this invertebrate model has been widely used to investigate the molecular properties and cellular functions of certain protein components of human viruses in the *in vitro* and *in vivo* platforms (**Table 1**) (Hughes et al., 2012; Panayidou et al., 2014) and to provide basic understandings on the conserved host antiviral immunity in the metazoan species (Xu and Cherry, 2014; Nainu et al., 2017). Among other aspects, the identification of host cellular factors required for SARS-CoV-2 replication is one of the important pillars that could provide valuable insights into novel targets for anti-COVID-19 therapy. An *in vitro* genome-wide RNA interference screen using *D. melanogaster* cells demonstrated the value of this approach to identify cellular host factors required for the replication of influenza A and dengue viruses (Hao et al., 2008; Sessions et al., 2009). In addition to that, a binary GAL4/UAS gene expression system in *Drosophila* was successfully used as an economical, rapid, and efficient *in vivo* platform to characterize the apoptotic role of two SARS-CoV proteins, protein 3a and M (Wong et al., 2005; Chan et al., 2007). The two-component GAL4/UAS system is comprised of the Gal4 gene, encoding the yeast transcription activator protein Gal4, and the UAS (Upstream Activation Sequence), a site to which GAL4 binds to activate the transcription of the transgene of interest downstream of the UAS (Brand and Perrimon, 1993). Using a similar approach, it would be reasonably feasible to closely study the function of SARS-CoV-2 genes in the context of whole *Drosophila* tissues *in vivo* or at different developmental stages.

**Table 1:** List of human viruses studied using *Drosophila* model system.

Genome type	Virus	Diseases in human	Experimental systems used in *Drosophila*	Lessons learned from *Drosophila* model	Refs
ssRNA (positive sense with RT)	Human immune-deficiency virus (HIV)-1	Acquired immune deficiency syndrome (AIDS)	*In vitro* (transfected cell culture), *In vivo* (transgenic fly to express viral proteins)	Inhibition of Toll pathway and induction of JNK pathway by HIV-1 Vpu are occurred in tissue-dependent manner	(Leulier et al., 2003; Marchal et al., 2012)
ssRNA (positive sense)	Dengue virus (DENV)	Dengue hemorrhagic fever (DHF) and dengue shock syndrome	*In vitro* genome-wide RNAi screen: *Drosophila* cell culture, infected with DENV serotypes 1-4	-The significance of RNAi to control DENV infection -Several host factors have been found to be important in the infection control. These factors are suggested to be conserved between *Drosophila* and humans.	(Sessions et al., 2009; Mukherjee and Hanley, 2010)
	severe acute respiratory syndrome coronavirus (SARS-CoV)	Atypical pneumonia	*In vivo* (transgenic fly to express viral proteins)	Possible interactions between the SARS-CoV 3a and M with cytochrome c and the AKT pathway of the host, respectively	(Wong et al., 2005; Chan et al., 2007)
	Sindbis virus (SINV)	Sindbis fever	*In vitro* and *in vivo* (natural infection)	The role of NRAMP family proteins in SINV entry into *Drosophila* (and mammalian cells) and the importance of ERK pathway in the intestinal immunity of *Drosophila* (and mosquito)	(Rose et al., 2011; Xu et al., 2013)
	West Nile virus (WNV)	West Nile fever (including meningitis and encephalitis)	*In vitro* and *in vivo* (natural infection)	Possible suppression of RNAi in *Drosophila* (and mammalian cells) by non-coding WNV RNA	(Chotkowski et al., 2008; Schnettler et al., 2012)
ssRNA (negative sense)	Influenza A virus (IAV)	Flu pandemics	*In vitro* genome-wide RNAi screen: *Drosophila* cell culture, infected with a genetically modified Influenza A virus	Several host factors have been found to be important in influenza virus replication and host cell programming. These factors are suggested to be conserved between *Drosophila* and humans.	(Hao et al., 2008)
	Vesicular stomatitis virus (VSV)	Flu-like illness; oncolytic virus	*In vitro* and *in vivo* (natural infection)	The role of *Drosophila* Toll-7 in autophagy induction (in a manner independent on the NF-κB activity) to limit VSV infection. This is similar to TLR7 role in mammals	(Nakamoto et al., 2012)
dsDNA	Epstein-Barr virus (EBV)	Infectious mononucleosis, several types of cancer, and multiple sclerosis	*In vivo* (transgenic fly to express viral proteins)	Identification of relevant human tumor suppressors that are targeted by the BRLF1 of EBV to induce tumorigenesis	(Adamson et al., 2005; Adamson and LaJeunesse, 2012)
	Human cytomega-lovirus (HCMV)	Birth defects	*In vivo* (transgenic fly to express viral proteins)	Potential inhibition of embryogenesis by viral proteins	(Steinberg et al., 2008)
dsDNA	Simian virus (SV) 40	Undecided (oncogenic role in tumor remains questionable)	*In vivo* (transgenic fly to express viral proteins)	Possible mechanism of oncogenesis by the small tumor antigen (ST) of SV40	(Kotadia et al., 2008)
	Vaccinia virus (VACV)	Rash and fever. Used as a vaccine for smallpox prevention	*In vitro* and *in vivo* (natural infection), *In vivo* (transgenic fly to express viral proteins)	Identification of host factors required viral entry	(Moser et al., 2010)

ss, single-stranded; ds, double stranded; RT, reverse transcriptase; RNAi, RNA interference.

Second, *D. melanogaster* retains many essential characteristics required in a model organism allowing for a straightforward, robust, and in-depth study of viral gene function in the *in vitro* and *in vivo* settings. Despite size differences, *Drosophila* shares around 75% genetic similarity with humans (Pandey and Nichols, 2011), allowing a robust genetically tractable approach to investigate the conserved function of viral proteins on host cells and host factor(s) required for a reproductive infection. With its relatively simple genetics, the fruit fly is highly amenable for possible genetic modifications, as demonstrated by an improved list of transgenic and mutant lines, including those designed using the latest CRISPR-based method applicable for human infectious disease models (Pandey and Nichols, 2011; Ugur et al., 2016). Moreover, *D. melanogaster* can be maintained easily and inexpensively in the laboratory, making it convenient to be used by researchers with limited funding (Pandey and Nichols, 2011). Its rapid propagation and short lifespan (approximately one month in length) (Pandey and Nichols, 2011) might serve as beneficial experimental traits that help reduce time to obtain results, which is certainly essential during the outbreak.

Undoubtedly, despite its numerous advantages, there exist several limitations that may pose a challenge to use *Drosophila* as a host model in human virus research. Of all, physiological and genetic differences between *Drosophila* and humans are two of the most anticipated constraints (Hughes et al., 2012; Panayidou et al., 2014). Nevertheless, it is worth noting that such limitations are typical for other animal models. Hence, a better way to study human viruses is to verify findings in *Drosophila* to other mammalian models as well as in humans. Alternatively, *Drosophila* can also be used as a complementary model system, together with other animal models, to understand antiviral immunity against COVID-19. For example, while the lack of adaptive immune responses in *Drosophila* has prevented its use in vaccine-related research, some of the human-homologous innate antiviral immunity had been first discovered or later confirmed in *Drosophila* (Xu and Cherry, 2014; Nainu et al., 2015), suggesting that fly research may reveal important insights in the innate immune responses against SARS-CoV-2. Besides, even though some mammalian viruses need to be modified before infection experiments in *Drosophila*, some of them can naturally enter *Drosophila* cells and are immediately detected by *Drosophila* immune factors and/or cells (see **Table 1**). This suggests the conserved infection mechanism(s) and innate immune responses between fruit flies and mammals. With this approach, we might provide some additional answers to remaining COVID-19-related questions in a reasonable, systematic, and economical manner.

Currently, *D. melanogaster* has been a leading model system for studying biochemical and biological aspects of human viruses and their pathogenic consequences on host cells (Hughes et al., 2012; Panayidou et al., 2014). The availability of sophisticated molecular tools for *in vitro* and *in vivo* experiments (Pandey and Nichols, 2011; Ugur et al., 2016), followed by the versatility of the model system and feasibility of research using human viruses (Hughes et al., 2012; Panayidou et al., 2014) are some of the powerful features in *Drosophila* that shall be beneficial to explore biological events in a precise detail that may be difficult to overcome using higher animal models. In the long run, we believe that *D. melanogaster* will serve as a convenient model organism for COVID-19-related research. For example, this model organism might help us to uncover factors related to host susceptibility to SARS-CoV-2 infection, as demonstrated in the case of influenza A and dengue viruses (Hao et al., 2008; Sessions et al., 2009), and whether those factors are clinically responsible in the human susceptibility to SARS-CoV-2. Alternatively, one may try to address the mechanistic basis of host innate immune activation in response to SARS-CoV-2 infection and whether disruptions in these mechanisms may yield different outcomes in the infected hosts. Certainly, with the right questions to ask, *D. melanogaster* would be a potential ally in the fight against COVID-19.

## Author Contributions

FN and DR designed and drafted the manuscript. FN, HH, and TE revised the manuscript critically for important intellectual content. All authors contributed to the article and approved the submitted version.

## Conflict of Interest

The authors declare that the research was conducted in the absence of any commercial or financial relationships that could be construed as a potential conflict of interest.
